# Targeting GJB4 to inhibit tumor growth and induce ferroptosis in pancreatic cancer

**DOI:** 10.3389/fonc.2025.1585236

**Published:** 2025-06-12

**Authors:** Xiangru Zheng, Wenjun Li, Xiaoli Li, Qianyi Yao, Luquan Zheng, Rongrong Fan, Ping Bie

**Affiliations:** ^1^ Department of Hepatobiliary and Pancreatic Surgery, The Third Affiliated Hospital of Chongqing Medical University, Chongqing, China; ^2^ Department of Pharmacy, The Third Affiliated Hospital of Chongqing Medical University, Chongqing, China; ^3^ Department of Pharmacology, College of Pharmacy, Chongqing Medical University, Chongqing, China; ^4^ Department of Nephrology, The Third Affiliated Hospital of Chongqing Medical University, Chongqing, China; ^5^ Department of Medicine Huddinge, Karolinska Institute, Stockholm, Sweden

**Keywords:** pancreatic cancer, GJB4, reactive oxygen species, ferroptosis, bioinformactics

## Abstract

**Background:**

Pancreatic cancer (PC) is a highly malignant type of cancer characterized by poor prognosis and high mortality rate. The role of gap junction protein beta 4 (GJB4) in PC has been scarcely reported. Therefore, the aim of this study was to investigate the function of GJB4 in PC cells and its potential as a therapeutic target.

**Methods:**

The expression of GJB4 in patients with PC was examined using data from The Cancer Genome Atlas (TCGA), The Human Protein Atlas, Gene Expression Omnibus (GEO), Tumor Immune Estimation Resource (TIMER), microRNA Target Prediction Database (miRDB), and Encyclopedia of RNA Interactomes (ENCORI). A short hairpin-RNA lentiviral vector was employed to downregulate GJB4 in PC cells. The effects on tumor cell proliferation, invasion, and metastasis were assessed using cell proliferation assays, Transwell migration assays, wound healing assays, and subcutaneous xenograft models. Bioinformatics analysis was conducted to explore the function of GJB4 in PC cells. Changes in ferroptosis in PC cells following GJB4 downregulation were detected by immunofluorescence staining, transmission electron microscopy, and western blotting.

**Results:**

GJB4 expression was found to be upregulated in PC tissues and positively correlated with patient survival. The expression of GJB4 was related to immune cell infiltration, tumor mutational burden expression and miRNAs. Downregulation of GJB4 inhibited the proliferation, metastasis, and invasion of PC cells, as well as tumor growth in nude mouse subcutaneous xenografts. Knockdown of GJB4 in PC cells disrupted oxidative and iron ion balance, and promoted ferroptosis, thereby inhibiting tumorigenic properties.

**Conclusion:**

The findings of this study indicate that downregulation of GJB4 may suppress tumor characteristics by promoting ferroptosis in PC cells. Therefore, GJB4 may be a promising therapeutic target for the treatment of PC.

## 1 Introduction

Pancreatic cancer (PC), a highly malignant type of cancer, is characterized by poor prognosis and high mortality rates (1). Despite early detection, prompt diagnosis, and standardized treatment, the probability of survival for patients with PC remains dismally low (2). This underscores the critical need for thorough research into the mechanisms underlying the development and progression of the disease to discover potential therapeutic targets.

Ferroptosis is a unique form of cell death, different from apoptosis, and is marked by oxidative damage to cell membranes (3). This process is initiated by perturbations in iron homeostasis, leading to the accumulation of ferrous ions (Fe2^+^) with potent oxidative capabilities (4). These ions trigger iron-dependent Fenton reactions, producing high levels of reactive oxygen species (ROS) inside cells (2). Subsequently, ROS cause oxidative damage to cell membranes, significantly increasing phospholipid hydroperoxides, particularly lipid hydroperoxides (5–7). The accumulation of lipid hydroperoxides within the cell ultimately leads to ferroptosis. Recent research indicates that inducing ferroptosis in tumor cells may be a promising strategy for improving the effectiveness of cancer drugs (8, 9). However, the use of ferroptosis in treating PC is still not widely established.

Gap junction proteins (connexins), encoded by the GJB gene family, comprise 21 homologs (GJB1-21). These are highly sensitive to microenvironmental alterations, which can significantly modulate their expression patterns (10–12). Given the well-established influence of the tumor microenvironment on cancer progression (13) and the critical role of gap junctions in facilitating intercellular communication – both among tumor cells and between tumor and immune cells (14) – connexins have emerged as important regulators in oncology.

The purpose of this study was to further elucidate the role of GJB4 in PC and to investigate the functional role of GJB4 and its underlying molecular mechanisms in ferroptosis. Our findings contribute to the understanding of the mechanisms underlying the potential of GJB4 as a therapeutic target for PC.

## 2 Materials and methods

### 2.1 Cell culture

The human PC cell lines PANC-1 (Catalog number: CL-0184, hereinafter referred to as PANC) and HPAC (Catalog number: CL-0361) were purchased from Wuhan Procell Life Science and Technology Co., Ltd. PANC cells were cultured in Dulbecco’s Modified Eagle Medium (Procell PM150210) containing 10% calf serum. HPAC was cultured in medium (Procell CM-0361) for HPAC cells.

### 2.2 Analysis of GJB4 expression in single-cell sequencing

The data were downloaded from the Gene Expression Omnibus (GEO) dataset (GSE154778) (15). Quality control and reduction of dimensionality were performed with R package Seurat (16). SingleR was used to annotate cell groups (17).

### 2.3 Immune cell infiltration investigation

Tumor Immune Estimation Resource (TIMER) (http://timer.cistrome.org/) was used to analyze the relationship between immune cells and GJB4 expression (18).

### 2.4 Tumor mutational burden expression with GJB4

R package maftools was loaded to map the waterfall plots of gene mutations in the high and low GJB4 expression groups, respectively (19). The data were downloaded from The Cancer Genome Atlas (TCGA) dataset by R package TCGAbiolinks (20).

### 2.5 Competing endogenous RNA network of GJB4

The relevant miRNAs were predicted by miRDB (https://mirdb.org/). The ENCORI database (https://rnasysu.com/encori/index.php) was used to analyze the correlation between miRNAs and long noncoding RNAs with GJB4 in pancreatic adenocarcinoma (PAAD) (21).

### 2.6 Steady knockdown of GJB4

A lentiviral system was used to knock down GJB4. The GJB4 knockdown sequence, shGJB4: 5’-GGGATTATGACATGCCCCGCG-3’, and negative control sequence, NC: 5’-GACTCGGCGTGCGCTGCATAA-3’, were inserted into the backbone plasmid pLVX-shRNA1 separately. The 293T cells were co-transfected with the backbone plasmid, pMD2.G, and psPAX2. Cell supernatants were collected and used to transfect PANC or HPAC cells. After transfection, puromycin was used to screen stable cell lines.

### 2.7 Propidium iodide assay

Cells were seeded into 24-well plates at a density of approximately 1×10^4^ cells per well. The next day, the medium was aspirated. Cells were washed once in 1× phosphate buffered saline (PBS) and incubated in 500 nM PI dye (Beyotime; ST511) at 37°C for 20 min. After removing the PI dye, the cells were washed thrice in PBS, fixed using 5% paraformaldehyde, and stained with 4’,6-diamidino-2-phenylindole (DAPI). Subsequently, images were captured by a fluorescence microscope (Nikon, Japan).

### 2.8 Calcein-acetoxymethyl assay

Calcein-AM solutions (Beyotime; C2013S) were incubated at 37°C for 30 min in the dark according to the instructions provided by the manufacturer. Images were captured by fluorescence microscopy (Nikon, Japan).

### 2.9 ROS assay

Cells were seeded into 24-well plates at a density of approximately 1×10^4^ cells per well. The next day, the medium was removed. The cells were washed once in 1×PBS, incubated in dihydroethidium (DHE) or dichloro-dihydro-fluorescein-diacetate (DCFH-DA) (10 μM) at 37°C for 20 min, and washed thrice in 1×PBS. Images were captured by fluorescence microscopy (Nikon, Japan).

### 2.10 Iron assay

The iron concentration in the cells was assessed using the Iron Content Determination Kit (Sigma–Aldrich LLC, Shanghai, China; MAK025) according to the instructions provided by the manufacturer. Cells (2×10^6^) were rapidly homogenized in iron test buffer and centrifuged to remove insoluble material. Samples (30 μL) were added to a 96-well plate and incubated with 5 μL of iron assay buffer (ferrous iron) or iron reducing agent (total iron) at 25°C to determine ferrous or total iron content, respectively. After 30 min, iron probe (100 μL) was added to each well, and the plate was incubated at 25°C for 60 min. Finally, the absorbance was determined with a microplate reader (BioTek, USA) at 593 nm.

### 2.11 Malondialdehyde assay

MDA content in cells was detected using the Lipid Peroxide MDA Assay Kit (Beyotime; S0131S). The test was performed according to the instructions provided by the manufacturer, and the absorbance was measured at 532 nm with a microplate reader.

### 2.12 Western blotting analysis

Cells were lysed using RIPA (Beyotime; P0013B) to obtain total cell protein. The protein concentration was determined by the BCA Protein Concentration Assay Kit (Beyotime; P0010). After mixing with 5× SDS-PAGE Sample Loading Buffer, samples were denatured at 100°C for 10 min. Next, the proteins were separated by electrophoresis in sodium dodecyl sulfate-polyacrylamide gel electrophoresis and transferred onto polyvinylidene difluoride (Millipore, Billerica, MA, USA) membranes. After blocking with 5% skim milk for 2 h at room temperature, the samples were incubated overnight at 4°C with the following primary antibodies purchased from Abmart: GJB4 (PS07587) and Proteintech: ferritin heavy chain (FTH; 11682-1-AP), transferrin receptor (TfRC; 65236-1-Ig), ferroportin (Fpn; 26601-1-AP), Cystine/glutamate transporter (xCT; 26864-1-AP), glutathione peroxidase 4 (GPX4; 30388-1-AP), ferroptosis suppressor protein 1 (FSP1; 20886-1-AP). Membranes were washed thrice with 1×tris-buffered saline with Tween 20 and incubated with the secondary antibody at room temperature for 2 h. Photographs were captured using the Odyssey^®^ CLx Imaging System (LI-COR Biosciences, USA) or the Tanon 5200 luminescence imaging system.

### 2.13 Transmission electron microscopy imaging

Knockdown and control PANC cells were seeded in 6-cm dishes and treated with RSL3 (0.1 μM) for 24 h. Cells were fixed with 2.5% glutaraldehyde and collected. Transmission electron microscopy imaging was performed at Lilai Biomedicine Experiment Center (Chengdu, China).

### 2.14 Mouse xenograft model

All animal procedures performed in this study were approved by the Animal Welfare Committee of Chongqing Medical University (Chongqing, China). To establish a tumor xenograft model, 5×10^6^ PANC cells were injected subcutaneously into the left hind leg. One week after injection, the body weight of the mice and tumor size were measured every 3 days. After 3 weeks, nude mice were executed by carbon dioxide asphyxiation, tumors were removed, and tumors were measured and weighed. Tumor tissues were embedded in paraffin and sectioned. Hematoxylin and eosin (H&E) (Hematoxylin and Eosin Staining Kit; Beyotime; C0105S) and Masson staining (Servicebio; G1006-20ML) were conducted based on the instructions provided by the manufacturer.

### 2.15 Statistical analysis

Statistical analyses were performed by R, version 4.4.1 and GraphPad 9.0.0. The differential expression between the two groups was analyzed by Student’s *t*-test, Wilcoxon signed-rank test, and Fisher’s exact test. The threshold of significant difference was set at *P*-values <0.05.

## 3 Results

### 3.1 High expression of GJB4 is associated with poor prognosis in patients with PC

**Figure 1:**
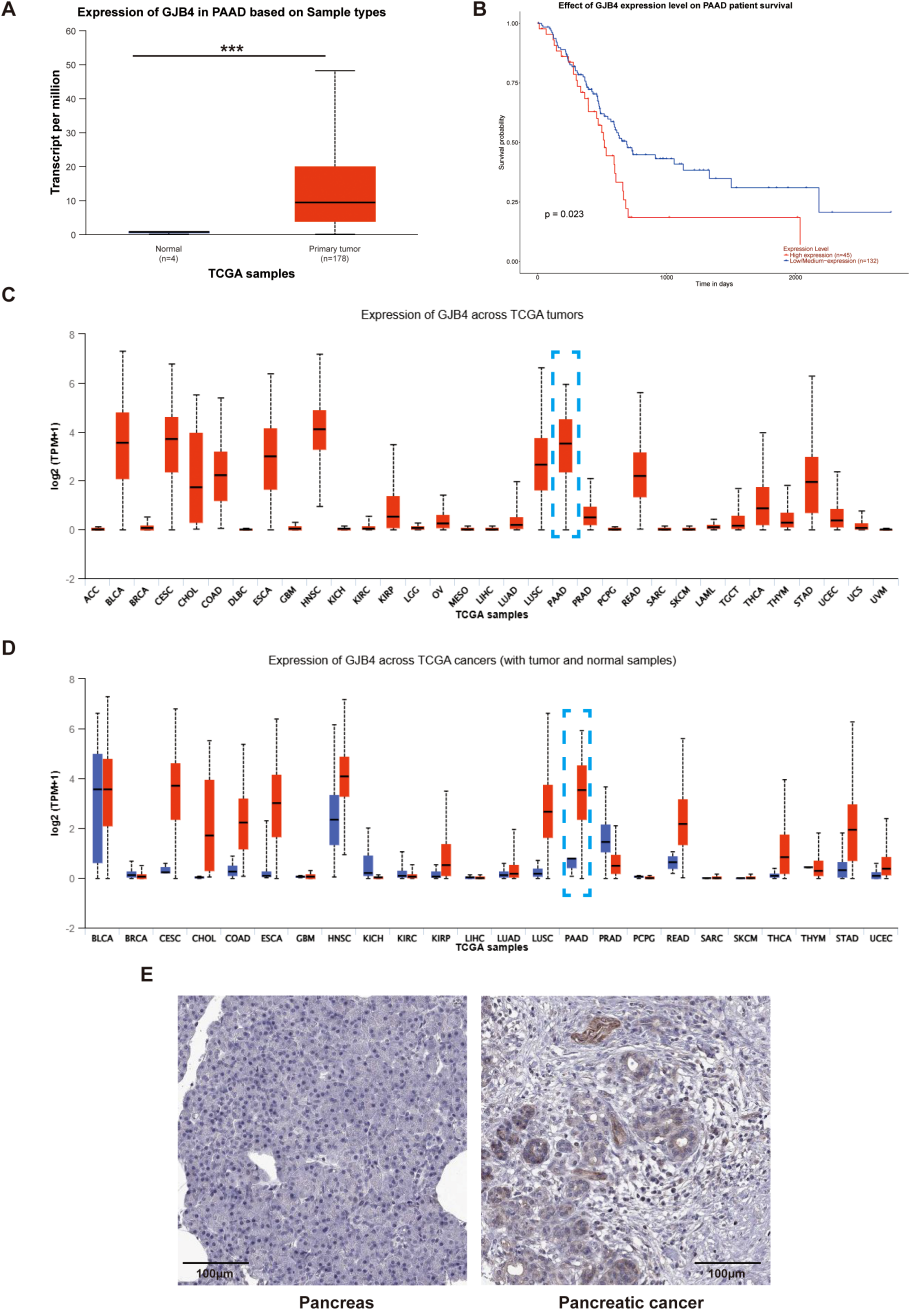
High expression of GJB4 is associated with poor prognosis in patients with PC. **(A)** GJB4 expression in PAAD in TCGA database. **(B)** Survival curves of patients grouped according to the expression of GJB4. **(C)** Expression of GJB4 in different tumors in TCGA database (blue box indicates PAAD). **(D)** Expression of GJB4 versus normal tissues in different tumors in TCGA database. **(E)** Expression of GJB4 in pancreatic cancer tumor tissue and normal pancreatic tissue in The Human Protein Atlas database. ***P < 0.001. GJB4, gap junction protein beta 4; TCGA, The Cancer Genome Atlas.

TCGA database was used to investigate the expression of GJB4 in PC tissues. In PC, the expression of GJB4 in cancerous tissues and adjacent tissues was significantly elevated (**Figure 1A**). Survival analysis showed that the survival curve of patients with high expression of GJB4 was lower than that of patients with low expression (**Figure 1B**). This finding suggests that patients with high expression of GJB4 had a shorter survival time. We investigated the expression of GJB4 in a total of 24 tumors included in TCGA database. GJB4 expression markedly varied in different tissues; for example, low expression was detected in normal tissues, such as liver hepatocellular carcinoma, thyroid carcinoma, and kidney renal papillary cell carcinoma, while high expression was observed in PAAD compared with other tumors (**Figures 1C, D**). These results suggest that the use of GJB4 as a therapeutic target for PC is associated with limited side effects on the liver and thyroid gland, as well as high tissue specificity. Subsequently, the expression of GJB4 in PC tumors was investigated in The Human Protein Atlas (https://www.proteinatlas.org/) database (**Figure 1E**). The data showed that GJB4 was highly expressed in PC and mainly expressed in PC tumor glandular tissues. These results suggest that GJB4 is associated with pancreatic tumor tissue glands. The differential expression of GJB4 in PC is obvious compared with other tumors. This result suggests that GJB4 has research value in PC.

### 3.2 Analysis of GJB4 expression across various cell types

**Figure 2:**
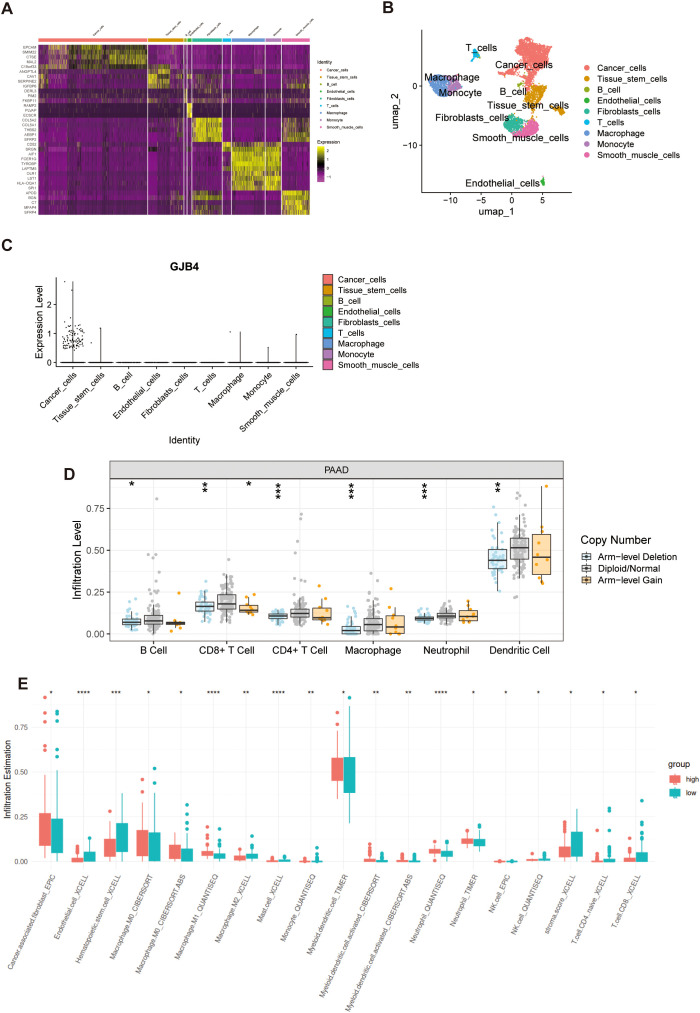
Single-cell and immune cell infiltration investigation of GJB4. **(A)** Heat map of the expression of marker genes. **(B)** UMAP map with cell annotations. **(C)** GJB4 expression in multiple cells. **(D)** The immune cell infiltration levels with different GJB4 CNV in PAAD. **(E)** Immune cell infiltration in the high and low GJB4 groups was analyzed using various computational methods. *P < 0.05, **P < 0.01, and ***P < 0.001. CNV, copy number variation; GJB4, gap junction protein beta 4; PAAD, pancreatic adenocarcinoma; GJB4, gap junction protein beta 4; TCGA, The Cancer Genome Atlas.

Single-cell sequencing data from PC cells (GSE154778) were downloaded, and SingleR was utilized for cellular type annotation. Following the processing methodology provided by the dataset developers (15), a heatmap was generated depicting the top 10 expressed genes across all cell types (**Figure 2A**). In accordance with the manuscript accompanying the dataset, epithelial cells were annotated as tumor cells (**Figure 2B**). Compared with all other cell types, GJB4 expression was observed exclusively in tumor cells (**Figure 2C**).

### 3.3 Immune cell infiltration based on the differential expression of GJB4

Samples in the arm-level deletion of GJB4 group showed decreased infiltration of immune cells, including B cells, CD8+ T cells, CD4+ T cells, macrophages, neutrophils, and dendritic cells (**Figure 2D**). Samples with high expression of GJB4 showed lower immune infiltration in seven immune cell groups (endothelial, hematopoietic stem cells, M2 macrophages, mast cells, monocytes, CD4 T cells, and CD8 T cells) and higher immune infiltration in six immune groups (cancer-associated fibroblasts, M0 macrophages, M1 macrophages, myeloid dendritic cells, activated myeloid dendritic cells, and neutrophils) (**Figure 2E**). In addition, the sample with high GJB4 expression had lower stromal scores (**Figure 2E**).

### 3.4 Differences in TMB between high and low GJB4 expression groups

**Figure 3:**
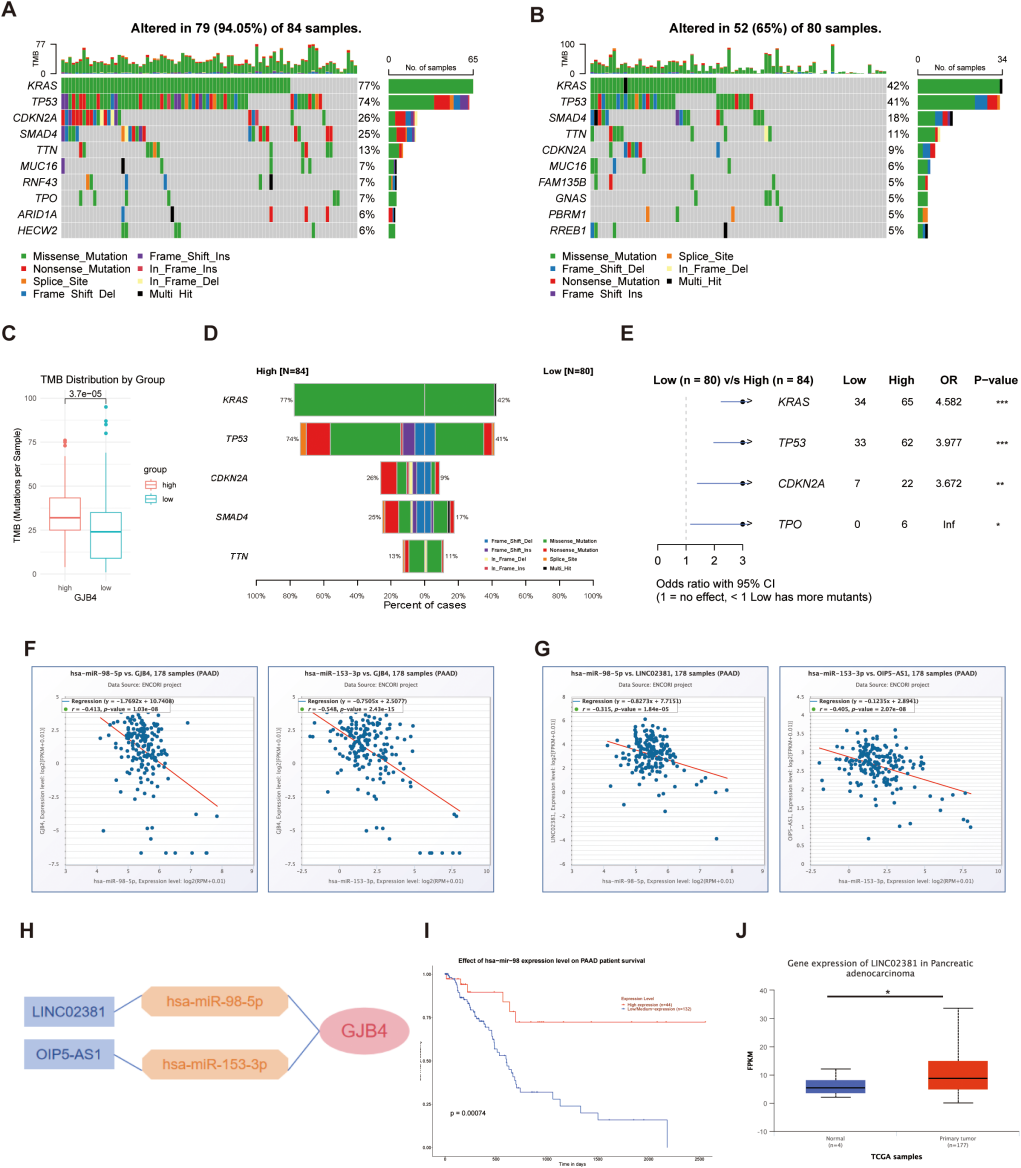
Investigating TMB and ceRNAs for GJB4. **(A, B)** Waterfall plots of genetic mutations in samples with high **(A)** and low **(B)** GJB4 expression. **(C)** Total TMB between the high and low GJB4 expression groups. Wilcoxon signed-rank test. **(D)** Comparisons between the high and low GJB4 expression groups. **(E)** Statistical results. Fisher’s exact test. **(F)** Correlation analysis between GJB4 and miRNA, has-miR-98-5p, and has-miR-153-3p. **(G)** Correlation analysis between has-miR-98-5p and has-miR-153-3p with lncRNA LINC02381 and OIP5-AS1, respectively. **(H)** The ceRNA regulation network. **(I)** Survival curve among patients with higher or lower has-miR-98 expression. **(J)** Different expression in patients with PAAD. *P < 0.05, **P < 0.01, and ***P < 0.001. GJB4, gap junction protein beta 4; TMB, tumor mutational burden, ceRNA, competing endogenous RNA; LINC02381, long intergenic non-protein coding RNA 2381; lncRNA, long noncoding RNA; OIP5-AS1, Opa interacting protein 5-antisense RNA 1; PAAD, pancreatic adenocarcinoma.

In patients with high expression of GJB4, 94.05% (79 of 84) exhibited tumor genetic mutations (**Figure 3A**). In contrast, among patients with low expression of GJB4, 65% (52 of 80) had genetic mutations (**Figure 3B**). Patients with high expression of GJB4 exhibited a higher TMB than those with low expression (**Figure 3C**). Specifically, within the high GJB4 expression group, 77% and 74% of patients had Ki-ras2 Kirsten rat sarcoma viral oncogene homolog gene and tumor protein p53 gene mutations, respectively. In the low GJB4 expression group, these values were 42% and 41%, respectively (**Figure 3D**). These differences were statistically significant (**Figure 3E**).

### 3.5 Investigating ceRNAs for GJB4

The predicted miRNAs has-miR-98-5p and has-miR-153-3p were negatively correlated with GJB4 (**Figure 3F**). The predicted long noncoding RNAs long intergenic non-protein coding RNA 2381 (LINC02381) and Opa interacting protein 5-antisense RNA 1 (OIP5-AS1) were negatively correlated with the expression levels of has-miR-98-5p and has-miR-153-3p, respectively (**Figure 3G**). The regulation network was predicted according to the co-expression relationships (**Figure 3H**). Among them, patients with higher has-miR-98 expression showed a better survival curve than other patients (**Figure 3I**), and LINC02381 was overexpressed in the PAAD group (**Figure 3J**).

### 3.6 Knockdown of GJB4 reduced the tumor phenotype of PC cells

**Figure 4:**
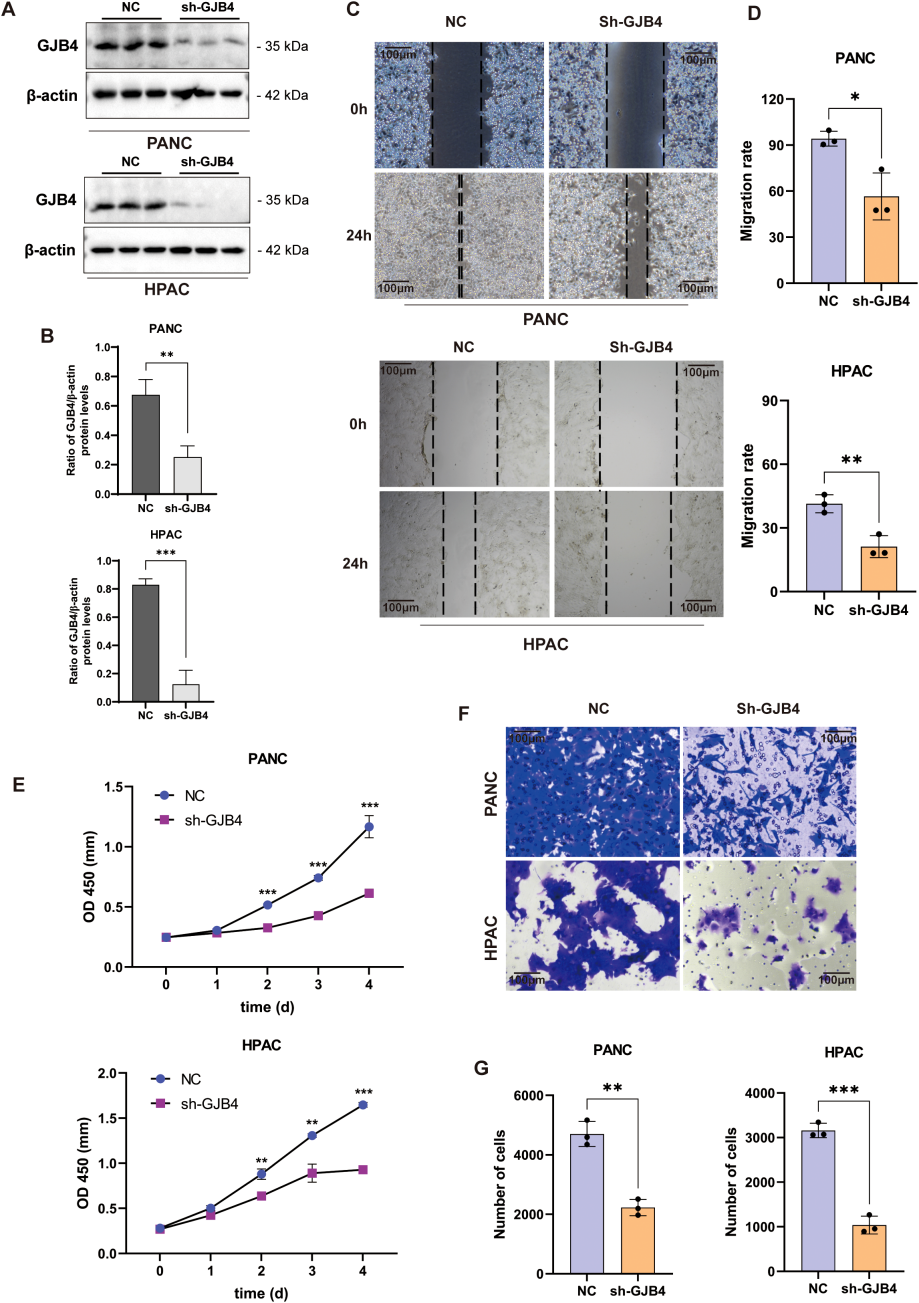
Knockdown of GJB4 reduced the tumor phenotype of PC cells. **(A)** WB detection of GJB4 expression in shGJB4-transfected PC cells. **(B)** WB grayscale chart. **(C)** Wound healing assay to detect the effect of shGJB4 on the migratory ability of PC cell lines. **(D)** Statistical chart of the results of the rate of migration. **(E)** CCK-8 assay to detect the effect of shGJB4 on PC cell proliferation. **(F)** Transwell assay to detect changes in the migratory ability of shGJB4-transfected cells. **(G)** Statistics of the Transwell experiment. *P < 0.05, **P < 0.01, and ***P < 0.001. CCK-8, Cell Counting Kit-8; GJB4, gap junction protein beta 4; WB, western blotting.

To investigate the function of GJB4 in PC, the lentiviral systems carrying shGJB4 and NC were introduced into PANC and HPAC cell lines, and the protein expression in the shGJB4 group was detected by WB. The results confirmed the knockdown of GJB4 protein in the cells (**Figures 4A, B**).

Subsequently, the effect of shGJB4 on the tumor phenotype was further examined. The changes in cell viability were continuously detected for four days with Cell Counting Kit-8 assay (**Figure 4E**), and the cell proliferation rate was observed. The results showed that the proliferation rate of the GJB4 knockdown group was slower than that of the control group, and the difference was statistically significant from the second day of observation.

The scratch assay was used to detect the effect of shGJB4 on the migratory ability of PC cells. At 24 h after scratching, PC cells in the shGJB4 group exhibited less healing than those in the control group (**Figures 4C, D**). The results suggested that the migratory ability of PC cells was reduced after transfection with shGJB4 and knockdown of GJB4. The Transwell experiment also revealed that the cells transfected with shGJB4 had reduced invasiveness compared with control group cells (**Figures 4F, G**). These data indicate that through enhance the proliferation and migration of PC cells, GJB4 can promote pancreatic tumor characteristics.

### 3.7 Knockdown of GJB4 inhibits the tumor phenotype of PC *in vivo*

**Figure 5:**
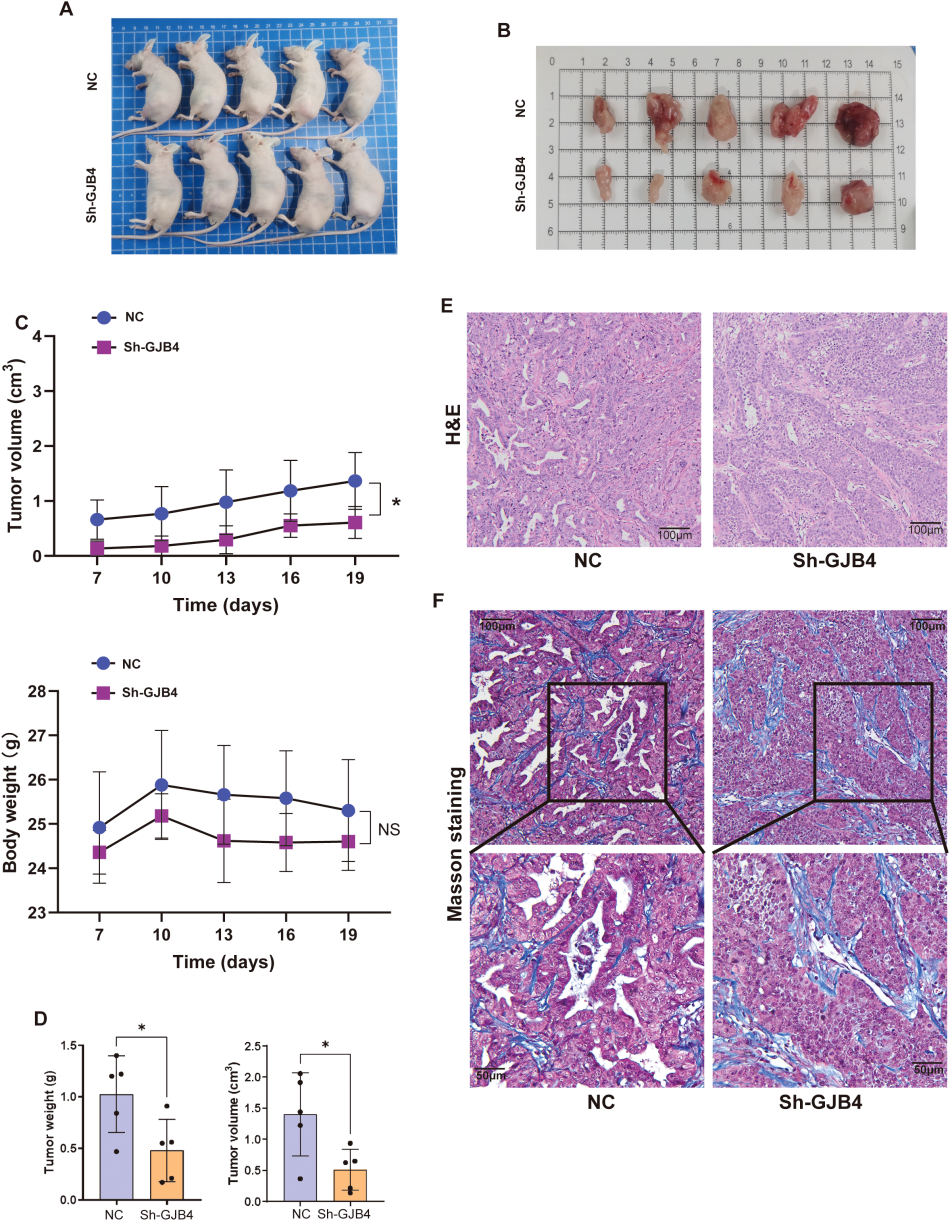
Knockdown of GJB4 inhibited the tumor phenotype of PC *in vivo*. **(A)** Cells transfected with shGJB4 or control vector were subcutaneously inoculated into the left flanks of nude mice. **(B)** Mice were sacrificed, and tumors were harvested. **(C)** The weight and volumes of harvested tumors were calculated. **(D)** The growth of tumors and body weight were measured once every 3 days. **(E)** H&E staining of harvested tumors. **(F)** Masson staining to distinguish tumor tissue from connective tissue. *P < 0.05. GJB4, gap junction protein beta 4; H&E, hematoxylin and eosin.

A mouse subcutaneous tumorigenesis model was used to evaluate the role of GJB4 *in vivo* (**Figures 5A, B**). The results showed that the tumor volume produced was significantly reduced in the shGJB4 group compared with the control group without statistically significant difference in body weight between the two groups (**Figure 5C**). Moreover, the volume and weight of shGJB4 tumors removed from the mice were reduced compared with those removed from the control group (**Figure 5D**). In addition, H&E staining revealed that the widely distributed gaps in the control group were reduced in the shGJB4 group (**Figure 5E**). To further distinguish whether these gaps are present in tumor cells or connective tissue, we performed Masson staining of tumor tissue (**Figure 5F**). The results showed that the cavity originally surrounded by tumor cells was significantly reduced in the shGJB4 group compared with the control group. This observation suggested that the original tumor structure of PC was destroyed after knockdown of GJB4, indicating the involvement of GJB4 in the structural formation of PC. These findings imply that knockdown of GJB4 reduces the tumor phenotype of PC *in vivo*.

### 3.8 Bioinformatics analysis suggested that GJB4 is associated with iron metabolism in PC

**Figure 6:**
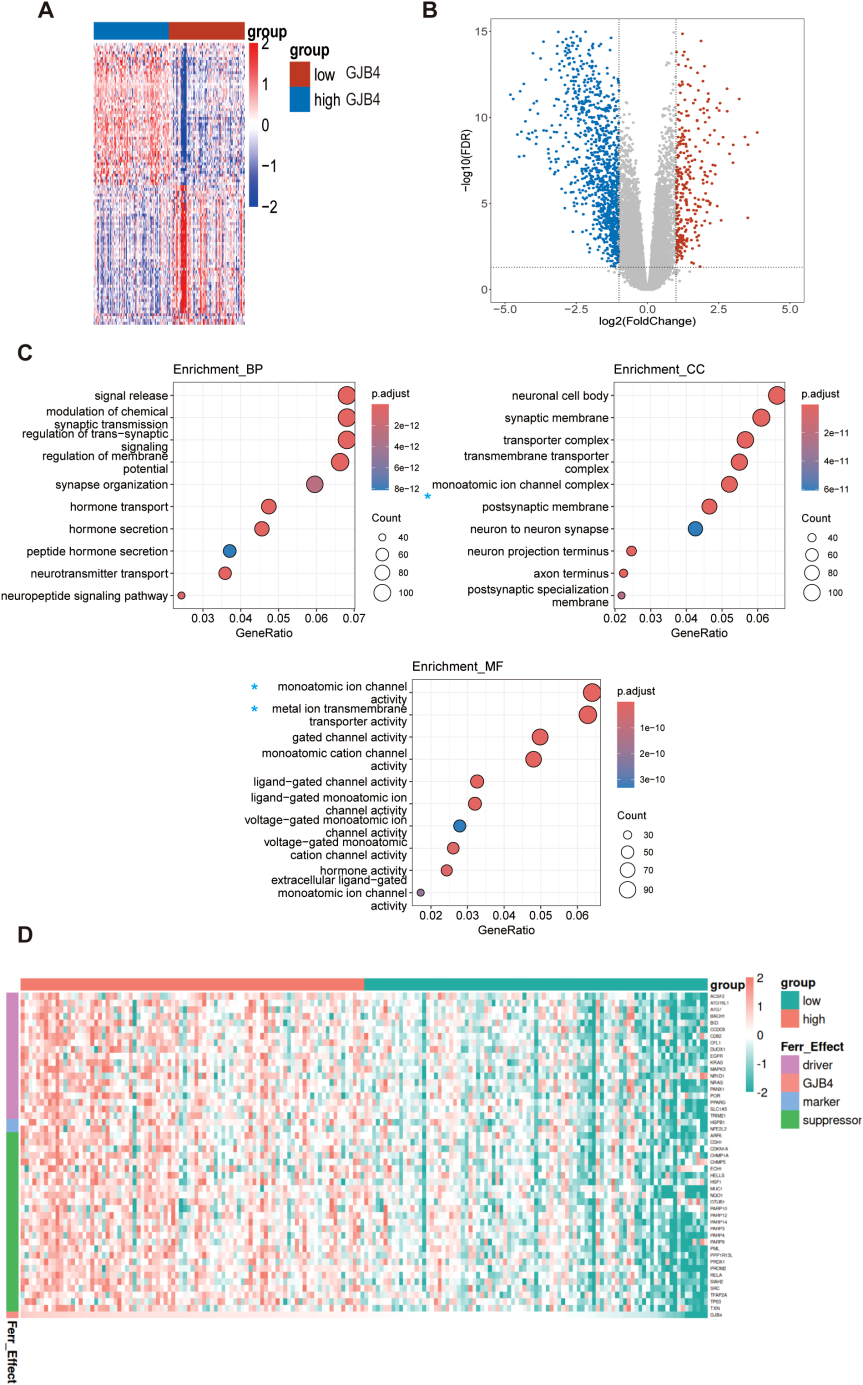
Bioinformatics analysis suggested that GJB4 is associated with iron metabolism in PC. **(A, B)** Differentially expressed gene heat maps and volcano maps of the high and low expression groups. **(C)** Enrichment analysis revealed that the differentially expressed genes were enriched in relation to the relevant iron metabolic pathway (blue asterisk). **(D)** Correlated gene heat map. A subset of genes, extracted from UALCAN and characterized by a Pearson correlation coefficient absolute value >0.3, corresponds to the ferroptosis genes recorded in FerrDb. The expression levels of these genes were utilized to construct the heat map. Driver, ferroptosis driver genes; Ferr_effect, ferroptosis effects; GJB4, gap junction protein beta 4; Marker, ferroptosis marker genes; Suppressor, ferroptosis suppressor genes.

To further investigate the mechanism through which GJB4 enhances tumor characteristics, we extracted the sequencing data of PAAD PC from TCGA database, divided the data into high and low expression groups based on the median expression of GJB4, and analyzed the differential gene expression between the two groups (**Figure 6A**). Differentially expressed genes with fold-change ≥1 and *P*-values <0.05 were selected for enrichment analysis (**Figure 6B**). In the analysis of cellular component, biological process, and molecular function (**Figure 6C**), we found that differentially expressed genes were enriched for multiple functions related to ion metabolism and transport, such as ion channel complex in cellular component and ion channel. Further analysis was conducted on genes extracted from UALCAN (22) that are associated with GJB4 expression. The study focused on genes with a Pearson correlation coefficient absolute value >0.3, which led to the identification of ferroptosis-related genes (as cataloged in FerrDb) (**Figure 6D**). We hypothesized that GJB4 may be associated with iron transport metabolism in PC cells. Recently, ferroptosis in PC has been identified as a key mechanism influencing the tumor characteristics of PC cells. Therefore, we suggest that GJB4 affects the phenotype of PC cells by regulating iron metabolism.

### 3.9 Knockdown of GJB4 promoted cell death and disrupted the balance of ROS and metal ions in PC cells

**Figure 7:**
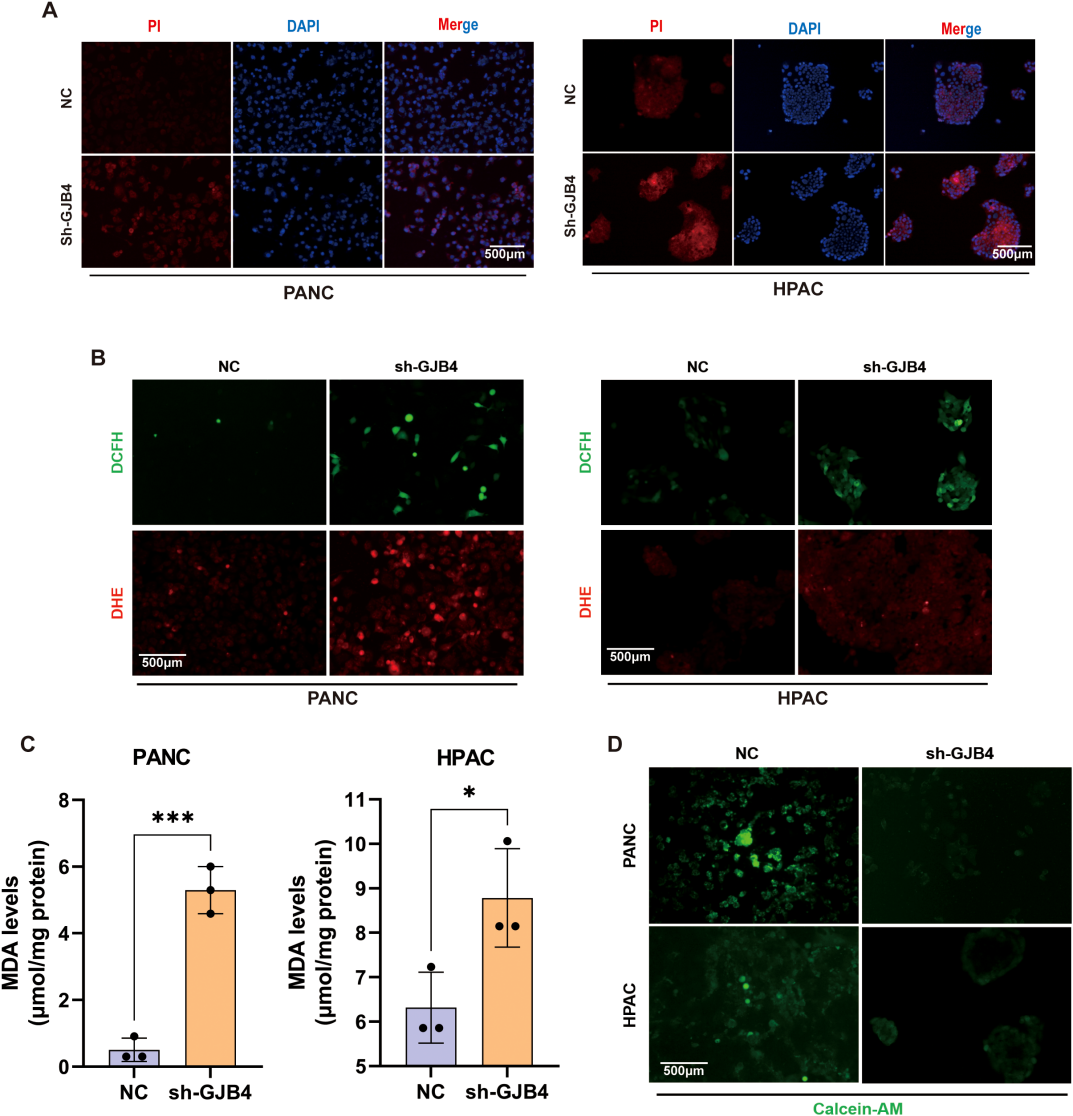
Knockdown of GJB4 promoted cell death and disrupted the balance of ROS and metal ions in PC cells. **(A)** Fluorescence intensity of PI staining was enhanced after knockdown of GJB4. **(B)** DCFH and DHE staining showed an increase in intracellular ROS after knockdown of GJB4. **(C)** MDA detection. **(D)** Calcein-AM staining. Knockdown of GJB4 and weakening of intracellular staining suggest that reducing the expression of GJB4 can disrupt the intracellular metal ion balance. *P < 0.05 and ***P < 0.001. Calcein-AM, Calcein-acetoxymethyl; DCFH, dichloro-dihydro-fluorescein-diacetate; DHE, dihydroethidium; GJB4, gap junction protein beta 4; MDA, malondialdehyde; PI, propidium iodide; ROS, reactive oxygen species.

To verify that GJB4 affects tumor cells by regulating ferroptosis in PC cells, we first examined the effect of GJB4 on PC cell death. PI staining assay was used to detect the effect of shGJB4 on cell death (**Figure 7A**). The results showed that the shGJB4 group had stronger fluorescence staining than the negative control group, indicating the presence of a greater number of dead cells. These results indicated that GJB4 may be involved in the regulation of cell death. Next, the concentration of endogenous ROS was detected using two immunoprobes, DHE and DCFH-DA (**Figure 7B**). The fluorescence intensity of DHE and DCFH-DA was higher in the shGJB group compared with the negative control group, indicating an increase in intracellular ROS concentration after knockdown of GJB4. These results suggest that GJB4 also stabilizes the role of intracellular ROS. Furthermore, the intracellular MDA content was also increased after knockdown of GJB4 (**Figure 7C**). Finally, the Calcein-AM was used to detect the concentration of intracellular metal ions (**Figure 7D**). The results showed that the fluorescence intensity of the shGJB4 group decreased, suggesting that the intracellular metal ions increased after knocking down GJB4. These results suggest that PC cell death, intracellular ROS, and metal ions increase after GJB4 knockdown, further denoting that GJB4 may regulate the tumor phenotypes of PC cells through ferroptosis.

### 3.10 Knockdown of GJB4 promotes ferroptosis in PC cells

**Figure 8:**
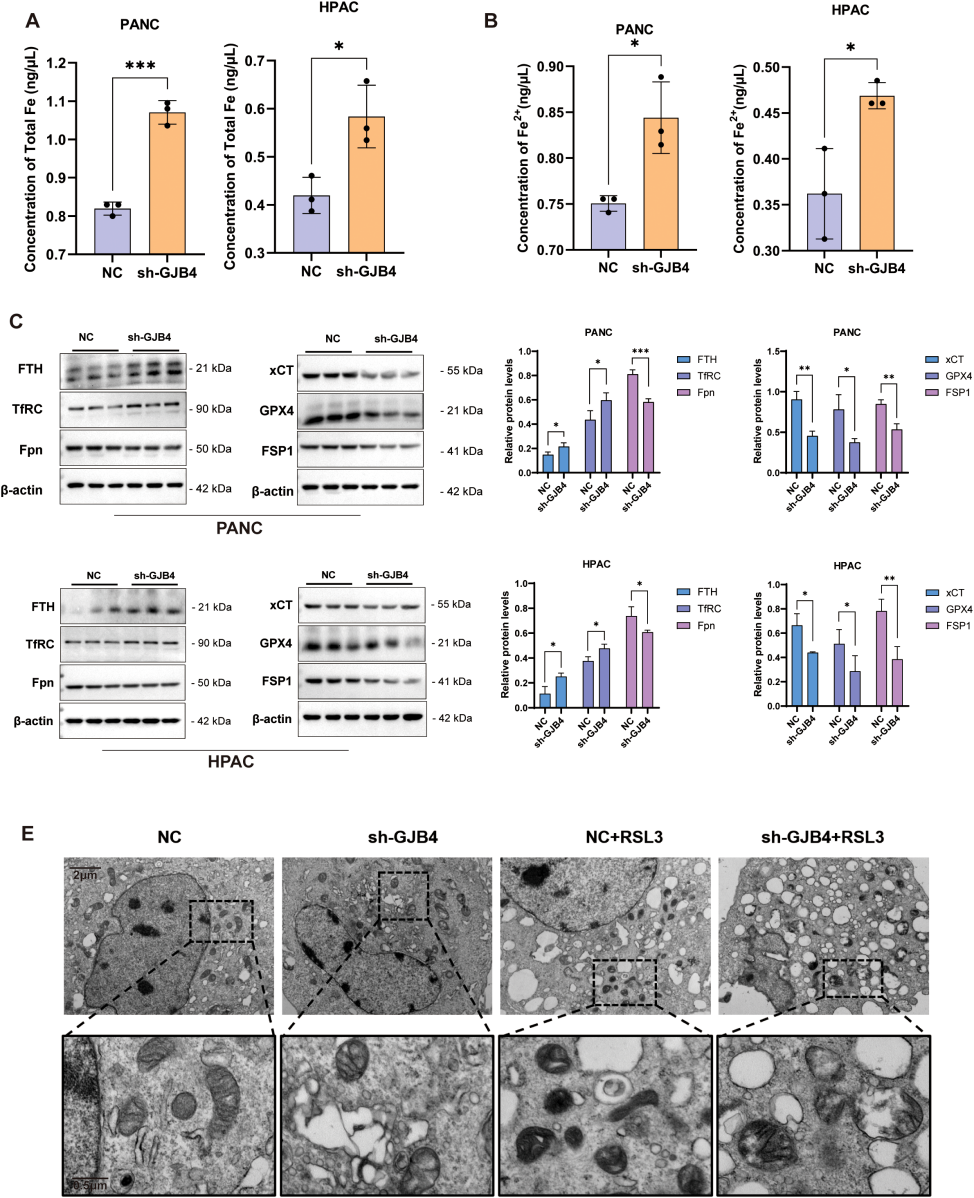
Knockdown of GJB4 promoted ferroptosis in PC cells. **(A, B)** Intracellular total iron content **(A)** and ferrous ion content **(B)** increased after knockdown of GJB4. **(C)** WB to detect ferritin changes. **(D)** Protein grayscale statistics. **(E)** Transmission electron microscopy to observe mitochondria within cells. *P < 0.05, **P < 0.01, and ***P < 0.001. GJB4, gap junction protein beta 4; WB, western blotting.

To further validate the effect of GJB4 on ferroptosis in PC cells, intracellular iron concentrations, including total iron and ferrous ion concentrations, were measured (**Figures 8A, B**). The results showed that both the total ferritin and ferrous ion concentrations increased significantly after knockdown of GJB4, suggesting that GJB4 could regulate the transport of iron ions in cells.

Subsequently, WB was used to detect the expression of iron transport-related proteins (**Figures 8C, D**). In the shGJB4 group, the expression of the protein FTH (which stores iron function) and the transferrin TfRC (which transfers iron from outside the cell) increased and decreased the expression of Fpn protein (which transfers iron ions out of the cell), indicating that the iron homeostasis of the cells was disrupted. WB detection of the core anti-ferroptosis protein of ferroptosis showed that the expression of xCT, GPX4, and FSP1 was reduced in the shGJB4 group, suggesting that intracellular ferroptosis increased after knockdown of GJB4.

Following the occurrence of ferroptosis, the mitochondrial volume of the cell decreases, the mitochondrial ridge decreases, the outer membrane is broken or thickened, the mitochondrial density increases, etc. (23, 24). The mitochondrial changes in PC cells PANC were detected by transmission electron microscopy, and the control cells were treated with the ferroptosis inducer RSL3 as a positive reference (**Figure 8E**). The results showed that the mitochondrial volume of the cells was reduced and the membrane was thickened by treatment with RSL3. In the shGJB4 group, the mitochondrial ridge became disordered; after adding RSL3, the mitochondrial was destroyed, suggesting that shGJB4 enhanced the effect of RSL3 on ferroptosis.

In summary, GJB4 knockdown can promote ferroptosis in PC cells. GJB4 maintains the tumor properties of PC cells by inhibiting ferroptosis.

## 4 Discussion

In this study, we found that high expression of GJB4 is associated with a poor prognosis in patients with PC. Knocking down GJB4 in PC cells inhibited the tumor phenotype of these cells. Bioinformatics analysis and subsequent experiments revealed that GJB4 was associated with iron metabolism in PC cells. Furthermore, the knockdown of GJB4 resulted in an increase in ROS and an enhanced susceptibility of PC cells to ferroptosis.

Members of the GJB family have important functions in a variety of tumors. GJB1 and GJB2 are highly expressed in glioblastoma and PC, which are closely related to chemotherapy resistance (25, 26). High expression of GJB1 and GJB2 was also associated with lymph node metastasis and prognosis in carcinoma of the pancreas (27). GJB2 has been shown to drive hepatocellular carcinoma progression by fostering an immunosuppressive tumor microenvironment (28). GJB1 enhanced the metastasis and invasion of lung cancer cells (29). GJB4, which has close relationship with tumors, increased the proliferation and metastasis of gastric cancer cells through Wnt/catenin beta 1 (Wnt/CTNNB1) (30). Moreover, it has been used as a tumor marker for lung cancer, because it promotes metastasis and chemotherapy resistance (31). In bladder cancer, GJB4 (target protein of miR-492) promoted the proliferation and metastasis of bladder cancer cells (32). In pancreatic tissue, GJB4 can increase insulin secretion (33). Consistent with our results, a recent study on GJB4 in PC also found a negative relationship with poor outcome of patients (26). Finally, our study suggests that GJB4 inhibited ferroptosis in PC cells, thereby preserving tumor properties. The present evidence enriches the understanding of the role of GJB family proteins in tumor diseases.

The treatment of tumors through the ferroptosis mechanism is increasingly being confirmed by research. For example, promoting ferroptosis in cells prevented the development of resistance to dabrafenib and trametinib during treatment, thereby enhancing the efficacy of melanoma therapy (23, 34). During immunotherapy, activated CD8^+^ T cells increased lipid peroxidation in tumor cells, which in turn promoted ferroptosis and enhanced the effectiveness of immunotherapy (35, 36). This approach targeted the tumor more effectively, and overcame the challenges associated with drug resistance and immune evasion (37). 2-imino-6-methoxy-2H-chromene-3-carbothioamide (IMCA) inhibited PC by inducing ferroptosis, indicating that suppression of ferroptosis pathways may be a major reason for the development of drug resistance in PC cells (38). Therefore, our findings on the molecular mechanisms of inhibition of ferroptosis in PC may provide a promising target for the treatment of PC.

GJB4 may cause ferroptosis by perturbing the tumor immune environment. In human tissue specimens, we discovered that the expression of GJB4 correlated with the location of PC glands. According to this result, the expression of GJB4 may be associated with the glandular structure. In the animal subcutaneous tumorigenesis experiment, we found that the tumor original cavity surrounded by tumor cells decreased in the GJB4 downregulation group, implying that GJB4 may be related to the tumor structure of PC. PC tumor structure is closely related to the tumor microenvironment (39). The conclusion that abnormalities in the cancer microenvironment cause ferroptosis in cancers (35, 40) hints that GJB4 caused ferroptosis in PC cells by disrupting the balance of the immune environment. In the future, we plan to investigate whether GJB4 changes tumor immunity by changing the tumor structure and altering the tumor environment.

## 5 Conclusions

GJB4 may be a therapeutic target for PC by regulating the molecular mechanisms in ferroptosis.

## Data Availability

The original contributions presented in the study are included in the article/supplementary material. Further inquiries can be directed to the corresponding author.
